# ALK-positive large B-cell lymphoma (ALK + LBCL) with aberrant CD3 expression

**DOI:** 10.1007/s12308-024-00582-x

**Published:** 2024-04-04

**Authors:** Jess Baker, Sara L. Zadeh, Nadine S. Aguilera

**Affiliations:** 1 Department of Pathology, University of Virginia Health System, Charlottesville, VA 22901-0214 USA; 2 Associated Pathologists PA, Tampa, FL USA

**Keywords:** Large B cell lymphoma, ALK expression, ALK rearrangement

## Abstract

ALK-positive ( +) large B cell lymphoma (ALK + LBCL) is a rare distinct subtype of diffuse large B cell lymphoma presenting with high stage and aggressive behavior. Although B cell markers such as CD20, CD19, and CD22 are generally negative, plasmacytic markers including CD138, CD38, and MUM1 are positive. T cell markers are negative with rare exceptions. We report an unusual case of ALK1 + LBCL in a 58-year-old man with partial expression of CD3 without other T cell antigen expression. The tissue was evaluated with flow cytometry, immunohistochemistry, fluorescent in situ hybridization, and gene rearrangement studies. Gene rearrangement studies for *IGH* and *TCR* gamma were performed. Flow cytometry did not demonstrate any abnormal lymphoid populations. Tissue sectioning shows a malignant plasmacytic large cell neoplasm which expresses CD45 but is negative for CD20, CD79a, and PAX5. Plasmacytic markers CD138 and MUM1 are positive with kappa light chain restriction. Strong granular cytoplasmic expression of ALK is present. FISH showing disrupted ALK supports the diagnosis while *MYC*, *BCL6*, and *BCL2* are intact*.* Gene rearrangement studies show coexisting *IGH* and *TCR* gamma clones; however, the *TCR* peak was present within a polyclonal background suggesting the disputed cells are likely only a subset of the T cell population. ALK + LBCL can present with an ambiguous immunophenotype, which warrants the use of multiple B cell, T cell, and plasmacytic antibodies. CD3 expression in this entity is rare and of uncertain clinical significance, but warrants further study.

## Introduction

Since the discovery of the oncogenic anaplastic kinase (ALK) gene in anaplastic large cell lymphoma [1–3], the number of ALK-positive neoplasms has expanded [4] and now includes ALK + LBCL, a rare subtype of aggressive diffuse large B cell lymphoma [5–10]. Architecturally, in lymph nodes, the neoplastic proliferation is diffuse with at least a partial sinusoidal pattern. Cell morphology shows plasmablastic or immunoblastic differentiation. Characteristically, immunohistochemistry in ALK + LBCL expresses plasmacytic markers including CD38, CD138, and MUM1. B cell and T cell markers are generally negative. ALK expression generally shows a cytoplasmic granular pattern. Although lineage-specific T cell markers are generally negative, up to 50% can show CD4 positivity [8, 9, 11]. Only rare cases are reported to express CD3 [9, 12]. Rare cases also have been reported to be EBV positive [13, 14].

The ALK protein is a receptor tyrosine kinase, encoded by the ALK gene on chromosome 2p23 [9]. Aberrant kinase activity associated with ALK can be seen with gene rearrangement, amplification, and point mutations of the ALK gene [4]; however, fusion proteins with ALK are seen most commonly in ALK + LBCL. ALK is rearranged in ALK + LBCL, most commonly *CLTC::ALK*, but other translocations have been reported involving the ALK gene [4–9, 11, 15, 16]. Multiple signaling pathways are stimulated in response to ALK, including Ras/ERK, JAK/STAT, PI3k/Ark, and PLCγ pathways [4]. Activation of STAT3 and STAT5 is reported in ALK + LBL [4] with STAT3 expression reported in some cases [17].

## Case history

A 58-year-old man presented with cervical adenopathy. The specimen was noted to be involved by a large cell neoplasm, which was favored to be hematopoietic, and referred to our institution. There is no other available past medical history or follow-up. Flow cytometry from an outside laboratory at the time of excision showed “no monotypic B-cell, phenotypically aberrant T-cell or blast cell population with identified.”

## Materials and methods

### Immunohistochemistry

Immunohistochemistry was performed according to standardized automated operating protocols. Immunohistochemistry included the following antibodies: CD45, ALK1, CD138, CD3, MYC, MUM1, EMA PAX5, CD79a, BCL6, CD10, CD21, BCL2, CD30, CD3, CD2, CD5, CD7, CD4, CD8, cyclin D1, CD56, HHV-8, P24 (HIV), AE1/AE3, MART-1, and in situ staining for Kappa, lambda, and EBER. CD3 performed at the outside institution was repeated at our institution using DAKO (A0452).

### T cell receptor (TCR) and immunoglobulin heavy (IGH) chain rearrangement

PCR-based detection for clonal TCR gamma and IGH was performed. Extracted DNA from the paraffin-embedded tissue using a modified version of QIAGEN QIAmp DNA purification protocol and the PCR-based BIOMED-2 assay (Invivoscribe, San Diego, CA) was performed. The manufacturer’s instructions were strictly followed in interpreting this assay. TCR beta was not performed.

### FISH

Deparaffinized tissue sections were hybridized using the manufacturer’s recommendations with the following probes Vysis LSI MYC Dual Color Breakapart DNA probe, Vysis IGH/BCL2 Dual Color Fusion DNA translocation probe, Vysis LSI BCL6 (ABR) Dual Color Breakapart DNA probe, and Vysis LSI ALK Dual Color Breakapart DNA probe.

## Results

**Fig. 1:**
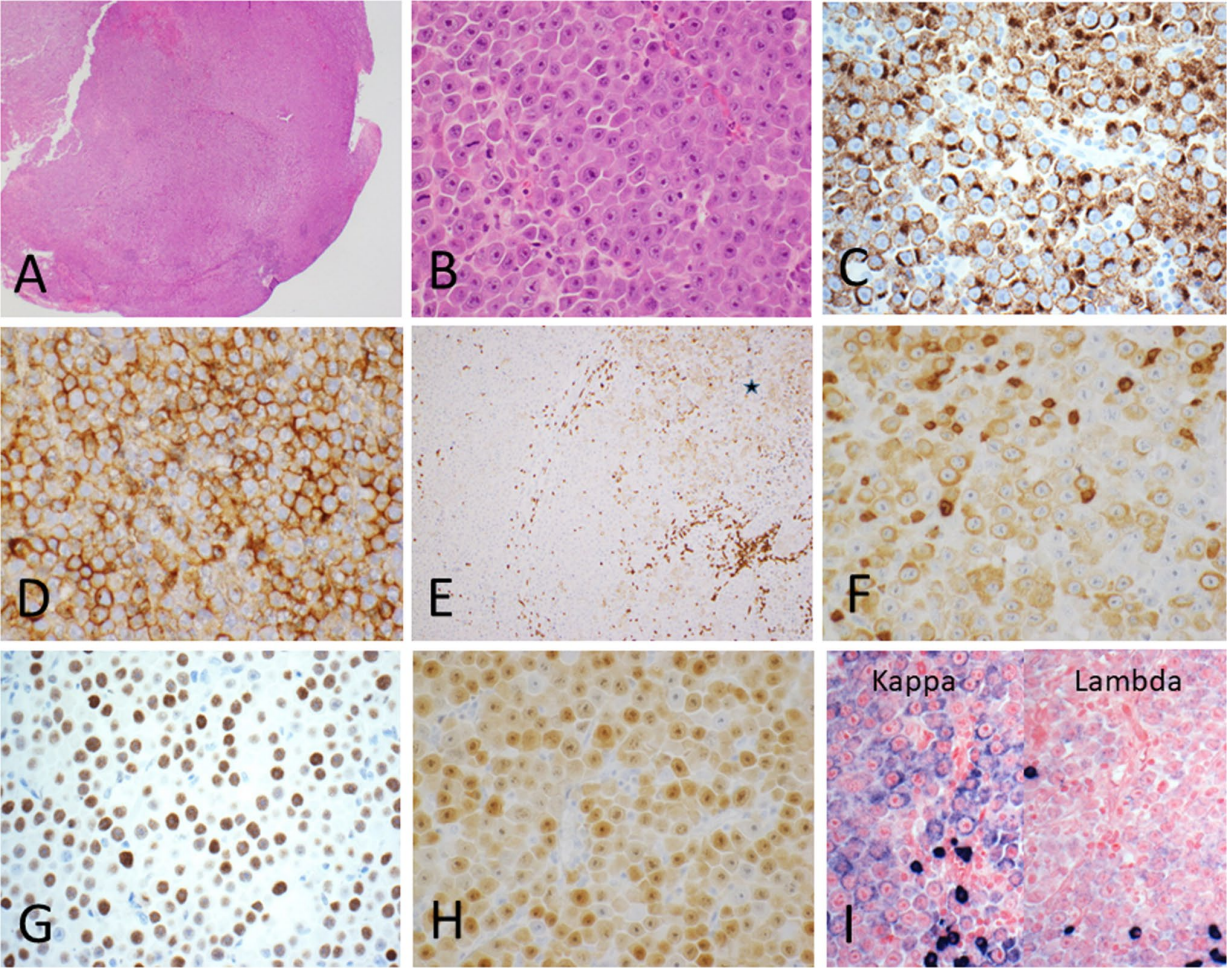
**A** Hematoxylin and eosin staining demonstrated lymph node tissue showing predominantly diffuse effacement of the architecture with a focal sinusoidal pattern (× 10 magnification). **B** A proliferation of large, atypical cells with nucleomegaly, round nuclear contours, prominent central macronucleoli, and plasmablastic features (× 400 magnification). **C** ALK1 shows granular cytoplasmic staining (× 400 magnification). **D** Positive CD138 (× 400 magnification). **E** Focal weak CD3 positivity (× 200 magnification). **F** High power of CD3 shows a cytoplasmic granular staining pattern (area of star in **E**) with small positive mature T cells showing strong membrane staining (× 400 magnification). **G** Positive MYC, greater than 40% (× 400 magnification). **H** Positive MUM1 (× 400 magnification). **I** Kappa (left) and lambda (right) showing kappa restriction in the neoplastic cells (× 400 magnification)

Hematoxylin and eosin staining demonstrated lymph node tissue showing predominantly diffuse effacement of the architecture (Fig. 1A) with a focal sinusoidal pattern. The sections show a proliferation of large, atypical cells with round nuclear contours, prominent central nucleoli, and plasmablastic features (Fig. 1B). Frequent mitotic figures and focal necrosis are present. Reported flow cytometry showed “no monotypic B-cell or aberrant T-cell population.”

Immunohistochemistry shows the neoplastic cells are positive for CD45, ALK1 (granular cytoplasmic staining) (Fig. 1C), CD138 (Fig. 1D), focal CD3 (Fig. 1E; star and 1F), MYC (Fig. 1G), MUM1 (Fig. 1H), and EMA (not shown). CD3 shows an unusual focal cytoplasmic and granular staining pattern in both the stain submitted by the outside institution and the repeated stain. In situ hybridization studies of kappa and lambda light chains demonstrate kappa restriction (Fig. 1I). Neoplastic cells are negative for CD20, PAX5, CD79a, BCL6 (weak to negative), CD10, CD21, BCL2, CD30, CD2, CD5, CD7, CD4, CD8, cyclin D1, CD56, HHV-8, P24 (HIV), AE1/AE3, MART-1, and in situ staining for EBER (not shown).

**Fig. 2:**
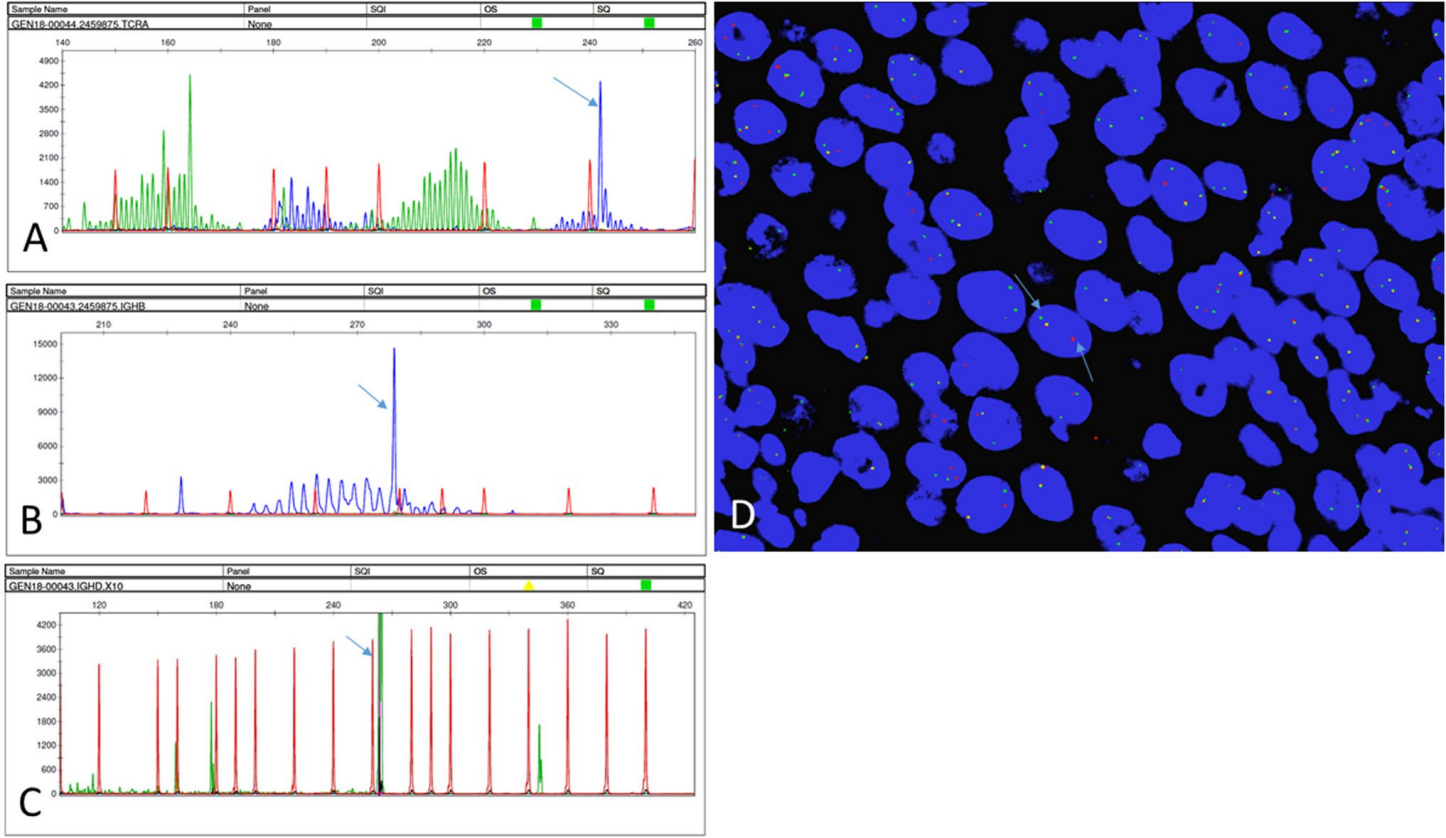
**A** TCR-PCR. The T cell receptor gamma-chain PCR assay demonstrates a predominant blue peak (arrow 242 bp) within a valid size range for tube A. **B** IGH-PCR. The immunoglobulin heavy chain gene PCR assay demonstrates a predominant black peak (arrow 279 bp) in tube B. **C** A predominant green peak (arrow 265 bp; 10 × dilution) in tube D. **D** Fluorescence in situ hybridization (FISH) for the ALK gene shows rearrangement (distinct green or orange signals at least one signal diameter apart (arrows)). The ALK partner cannot be determined with this FISH assay

Fluorescence in situ hybridization (FISH) reveals intact *MYC*, *BCL6* genes, and negative *IGH::BCL2*. ALK is disrupted (Fig. 2D). Clonal *IGH* and *TCR* gamma chain gene rearrangements are present (Fig. 2A–C). The *TCR* gamma shows a prominent peak in an otherwise polyclonal background.

## Discussion

ALK + LBCL is a distinct, rare, and aggressive type of large B cell lymphoma, which typically presents in lymph nodes, most commonly the cervical [7, 8, 11, 15, 16]; however, extranodal sites are also affected. There is a wide age range at presentation with a median of 35–38 years and a male predominance. Patients present with high stage disease in most cases with median survival of 11–24 months [4, 6, 8]. There is no known association with immunodeficiency or viral association including Epstein Barr virus, HHV8, or HIV. Architecture of the lymph node is diffusely effaced but often shows a partial sinusoidal pattern. Cellular morphology appears immunoblastic and/or plasmablastic [7, 8, 11, 15]. Rarely large Reed-Sternberg-like cells can be present [7]. There is increased mitotic activity and can be necrosis. Our case showed effaced architecture with the neoplastic cells showing a plasmablastic appearance.

Immunohistochemistry evaluation of the neoplastic cells in ALK + LBCL shows expression of plasmacytic markers including CD138, MUM1, CD38, and VS38. CD20 is often negative or weakly positive in a minority of cases. Other B cell antibodies including CD19 and CD22 are negative. PAX5 and CD79a are seen in a minority of cases. Other stains, which may be positive, include EMA, BOB-1, OCT-2, and CD45RB (may be weak). Cytoplasmic immunoglobulin most often IgA can be positive. CD10, MYC, and STAT3 can be positive. Lineage-specific T cell antigens and T cell-associated antigens are generally negative; however, CD4, CD57, and CD43 expression is reported in approximately half of cases. Immunohistochemistry in our case was compatible with ALK + LBCL, but the cells demonstrated partial CD3 expression, a finding that has been reported in only rare cases [9, 12]. EBV is almost uniformly negative, although rare cases have been positive [14]. CD30 is negative in the majority of cases.

All cases express ALK protein or contain the ALK translocation [9]. The staining pattern of ALK is most often in a cytoplasmic granular pattern; this expression pattern is the result of the lymphomagenic translocation *ALK::CLTC* [t(2;17)(p23;q23)] in the majority of cases. Other translocation partners include *NPM1*, *SEC31A*, *SQSTM1*, *RANBP2 GORASP2*, *EML4*, and *IGL*; these translocations can give distinct staining patterns [7]*.* Our case shows cytoplasmic granular ALK staining. ALK by FISH was disrupted in our case; however, the partner gene cannot be determined in the FISH assay and NGS was not performed. FISH for *BCL2*, *BCL6*, and *MYC* were intact.

Aberrant T cell-specific markers expressed on LBCL have been reported, most notably CD5, but other markers including CD2, CD4, CD7, and CD8 have also been reported [18, 19]. Aberrant CD3 on LBCL is unusual but is reported sporadically [18–22]. Some cases with CD3 expression on LBCL have been associated with EBV [13] leading to speculation that EBV may promote linage infidelity; however, EBV is not uniformly present, and our case does not express EBER. Expression of more than one T cell-specific or associated marker is seen in rare cases of LBCL. The biologic significance is unknown in cases of LCBL with aberrant CD3 due to their rarity [13, 19, 20].

ALK + LBCL has not been studied extensively with *IGH* and *TCR* previously, but in studies where IGH and TCR rearrangements were performed, no *TCR* clonal rearrangement was identified; however, no case showed CD3 expression in these studies [8, 11]. Coexisting *IGH* and *TCR* clones in gene rearrangement studies is not previously reported, but there are limited studies looking at *TCR* rearrangements in ALK + LBCL. Although our case may be considered “borderline” in some laboratories due to the apparent polyclonal background, the TCR assay was read as clonal following the manufacturer’s instructions. The source of this peak may derive from the focal neoplastic cells, which express aberrant CD3 or conversely, the peak may derive background T cells reacting to the neoplastic B cells. It has also been documented that false positive TCR rearrangements can occur in B cell lymphomas and reactive processes using BIOMED-2 assays [23, 24]. Although theoretically TCR gamma and TCR beta assays together could be used to confirm the clone, both may sometimes be clonal in the same reactive process [23]. Although the source and significance of the T cell peak in our case is uncertain, the utility of BIOMED-2 assays is well documented, and this assay is standard worldwide but caution in reading the assay should be observed.

In conclusion, we present a case of ALK + LBCL with aberrant expression of CD3 and gene rearrangement of both *IGH* and *TCR*. A panel of immunohistochemistry markers including multiple T cell, B cell, and plasmacytic markers was necessary to characterize the lymphoma, which is recommended when lineage-specific antigen expression is ambiguous. Because aberrant expression of CD3 is rare in ALK + LBCL, we performed *IGH* and *TCR* to help delineate lineage of this ALK + LBCL, testing which may be helpful particularly if aberrant T cell markers are expressed. However, as this case illustrates, gene rearrangement studies should be interpreted with caution and considered within the clinical and pathologic context. The clinical behavior of our case of ALK + LBCL is uncertain due to the rarity of aberrant CD3 in this entity and the lack of clinical follow-up information. The clinical behavior may not differ from conventional cases of ALK + LBCL; however, further study is necessary.
